# Imprints of massive black-hole binaries on neighbouring decihertz gravitational-wave sources

**DOI:** 10.1038/s41550-024-02338-0

**Published:** 2024-08-05

**Authors:** Jakob Stegmann, Lorenz Zwick, Sander M. Vermeulen, Fabio Antonini, Lucio Mayer

**Affiliations:** 1 Max Planck Institute for Astrophysics, Garching, Germany; 2 Gravity Exploration Institute, School of Physics and Astronomy, Cardiff University, Cardiff, UK; 3 Niels Bohr International Academy, Niels Bohr Institute, Copenhagen, Denmark; 4 Center for Theoretical Astrophysics and Cosmology, Institute for Computational Science, University of Zurich, Zurich, Switzerland; 5 California Institute of Technology, Department of Physics, Pasadena, CA USA

**Keywords:** Astronomical instrumentation, Compact astrophysical objects, General relativity and gravity

## Abstract

The most massive black holes in our Universe form binaries at the centre of merging galaxies. The recent evidence for a gravitational-wave (GW) background from pulsar timing may constitute the first observation that these supermassive black-hole binaries (SMBHBs) merge. Yet, the most massive SMBHBs are out of reach of interferometric GW detectors and are exceedingly difficult to resolve individually with pulsar timing. These limitations call for unexplored strategies to detect individual SMBHBs in the uncharted frequency band ≲10^−5^ Hz to establish their abundance and decipher the coevolution with their host galaxies. Here we show that SMBHBs imprint detectable long-term modulations on GWs from stellar-mass binaries residing in the same galaxy at a distance *d* ≲ 1 kpc. We determine that proposed decihertz GW interferometers sensitive to numerous stellar-mass binaries could uncover modulations from ~*O*(10^−1^–10^4^) SMBHBs with masses ~*O*(10^7^–10^8^) M_⊙_out to redshift *z* ≈ 3.5. This offers a unique opportunity to map the population of SMBHBs through cosmic time, which might remain inaccessible otherwise.

## Abstract (web summary)

Merging supermassive black holes emit low-frequency gravitational waves, difficult to observe with current and future detectors. Stegmann et al. show that these black holes can leave measurable traces in high-frequency signals from adjacent sources.

## Main

**Fig. 1:**
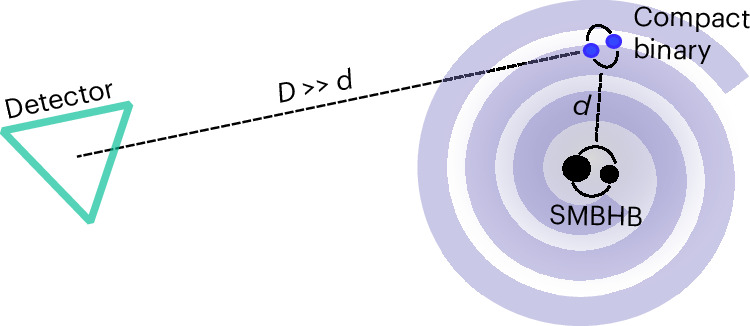
Cartoon of the method proposed in this work. The presence of an SMBHB emitting GWs causes frequency modulations in the GW emission of a compact binary source at a distance *d*. The modulations can be observed over a long observation time *T* with proposed decihertz GW detectors, at a distance *D* ≫ *d*. We show how this scenario would allow decihertz detectors to indirectly probe the existence of SMBHBs within the ~10^7^–10^9^ M_⊙_ mass range.

We consider the situation in which we directly detect a GW signal from a stellar-mass compact binary at luminosity distance *D* that is accompanied by an SMBHB in its proximity at *d* ≪ *D*, for example, at the centre of its galaxy (Fig. 1). The GWs emitted by the SMBHB perturb spacetime at the location of the compact binary, which induces a small frequency modulation in the detected signal (cf. equation (11)):

1 $${f}_{{{{\rm{measured}}}}}(t)\approx f(t)-f(t)\zeta (t)+{{{\mathcal{O}}}}({\zeta }^{\,2}),$$

where *f*(*t*) is the unmodulated frequency of the compact binary and *ζ*(*t*) ≪ 1 is a small modulation. At the lowest order, the SMBHB causes a monochromatic modulation that can be written as a sinusoid (cf. equation (14)):

2 $$\zeta (t)={{{{\mathcal{A}}}}}_{{{{\rm{mod}}}}}\cos (2\uppi {f}_{{{{\rm{mod}}}}}t+{\phi }_{{{{\rm{mod}}}}}),$$

where ${f}_{{{{\rm{mod}}}}}$ is the frequency of the modulating GW and its amplitude ${{{{\mathcal{A}}}}}_{{{{\rm{mod}}}}}$ is of the order $\sim {(\pi {f}_{{{{\rm{mod}}}}})}^{2/3}{{{{\mathcal{M}}}}}_{{{{\rm{mod}}}}}^{5/3}/d$, with ${{{{\mathcal{M}}}}}_{{{{\rm{mod}}}}}$ being the chirp mass of the SMBHB. Thus, the detector receives modulated GWs from the compact binary (cf. equation (21)), where the modulation is fully described by three additional physical parameters that are determined by the SMBHB: an overall amplitude ${{{{\mathcal{A}}}}}_{{{{\rm{mod}}}}}$, a modulating frequency ${f}_{{{{\rm{mod}}}}}$ and a phase ${\phi }_{{{{\rm{mod}}}}}$.

**Fig. 2:**
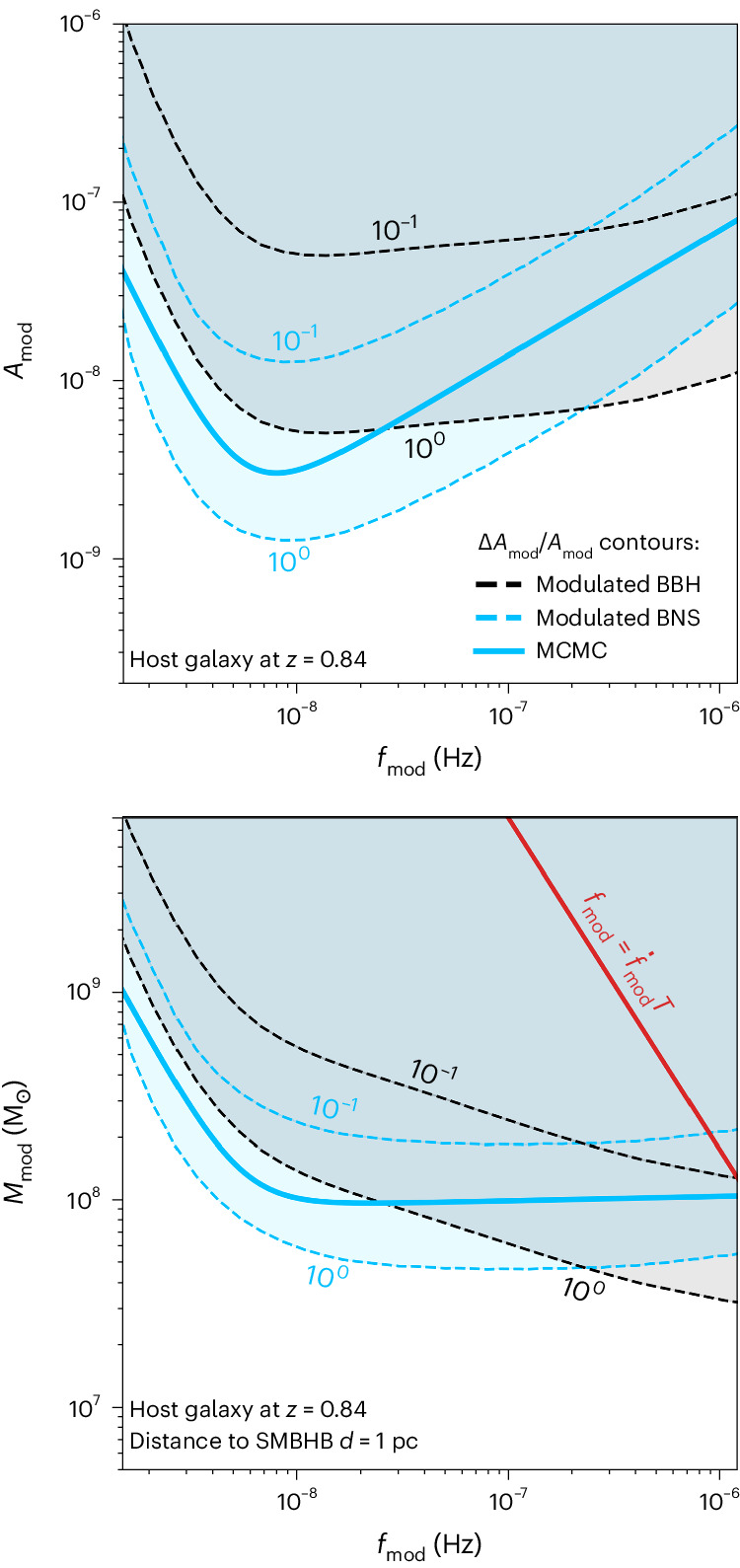
Detectability contours of the modulating SMBHB amplitude ${{{{\mathcal{A}}}}}_{{{{\rm{mod}}}}}$ experienced by a stellar-mass compact binary. The binaries are located in a fiducial host galaxy at redshift *z* = 0.84. The modulated GW signal from the compact binary is observed with DECIGO over *T* = 10 years. Left: to compute this sensitivity we vary the modulating amplitude ${{{{\mathcal{A}}}}}_{{{{\rm{mod}}}}}$ and frequency ${f}_{{{{\rm{mod}}}}}$ of the SMBHB and indicate the relative uncertainty $\Delta {{{{\mathcal{A}}}}}_{{{{\rm{mod}}}}}/{{{{\mathcal{A}}}}}_{{{{\rm{mod}}}}}=1{0}^{0}$ and 10^−1^, respectively, by which a given amplitude could be recovered with dashed contour lines, using parameter estimation with the Fisher matrix. Black contour lines assume that a GW150914 (ref. ^100^)-like BBH with a chirp mass of ${{{\mathcal{M}}}}=28.0\,{{{{\rm{M}}}}}_{\odot }$ is observed. Blue contour lines assume a GW170817 (ref. ^68^)-like BNS (${{{\mathcal{M}}}}=1.188\,{{{{\rm{M}}}}}_{\odot }$). We also show for the BNS the sensitivity curve from a full Bayesian analysis with an MCMC (solid blue line) that corresponds to the Fisher matrix uncertainty $\Delta {{{{\mathcal{A}}}}}_{{{{\rm{mod}}}}}/{{{{\mathcal{A}}}}}_{{{{\rm{mod}}}}} \approx 1{0}^{-1/2}$ (Methods and Supplementary Fig. 2). Right: we show the sensitivity in terms of the minimum detectable chirp mass ${{{{\mathcal{M}}}}}_{{{{\rm{mod}}}}}={({{{{\mathcal{A}}}}}_{{{{\rm{mod}}}}}d)}^{3/5}/{(\uppi {f}_{{{{\rm{mod}}}}})}^{2/5}$ of the SMBHB for a fiducial distance *d* = 1 pc to the BBH/BNS. The red line indicates the frequency above which our assumption of a monochromatic SMBHB breaks down. Note that the number of SMBHBs near this limit is anyways highly suppressed (Methods).

To investigate how well these additional parameters can be measured, we employ two methods. First, we calculate the Fisher matrix of the modulated post-Newtonian (PN) waveform of the compact binary that includes both higher-order modes and effective spin (Methods). The Fisher matrix approach allows us to efficiently survey a large part of the parameter space and to estimate the expected error of fitting the parameters of the sinusoidal modulation to the noisy data of a detector (Methods). Second, we run a series of full Bayesian parameter recovery tests employing numerical Markov chain Monte Carlo (MCMC) methods at a representative redshift of *z* = 0.84. In the latter method, we explore a large grid of injected values of ${{{{\mathcal{A}}}}}_{{{{\rm{mod}}}}}$ and ${f}_{{{{\rm{mod}}}}}$ to find the curve delimiting detectable and undetectable modulations, where we consider a modulation to be detectable if the one-sigma width of the posterior distribution of ${{{{\mathcal{A}}}}}_{{{{\rm{mod}}}}}$ is smaller than the injected value. We find that this ensures that the detectable modulations are distinguishable from the null hypothesis of no modulation (${{{{\mathcal{A}}}}}_{{{{\rm{mod}}}}}=0$) being present with extremely high confidence (see Methods for a more thorough discussion). Finally, as seen in Fig. 2, we can match the curve defined by this strict criterion to a corresponding relative error $\Delta {{{\mathcal{A}}}}_{{{\rm{mod}}}}/{{{\mathcal{A}}}}_{{{\rm{mod}}}}\equiv \sqrt{\langle {(\Delta {{{\mathcal{A}}}}_{{{\rm{mod}}}})}^{2}\rangle }/{{{\mathcal{A}}}}_{{{\rm{mod}}}}\lesssim 1{0}^{-1/2}$ that results from the simpler Fisher matrix analysis. For the purposes of numerical efficiency, we use the scaling with redshift of the latter to extrapolate the stricter MCMC criterion.

Modulations caused by SMBHBs can be most easily identified with a detector that can observe a large number of compact binaries with a high signal-to-noise ratio (SNR) and over a long observation time *T*, which is limited by the mission lifetime of the detector. We find that these constraints single out decihertz GW detectors^1^ and the large populations of binary black holes (BBHs) and binary neutron stars (BNSs) that they are expected to observe. Thus, we adopt the sensitivity curves of the proposed space-based laser-interferometric GW detectors DECIGO and BBO^2,3^ as examples for any sufficiently sensitive detector in the decihertz regime. In Fig. 2, we showcase the results of our Fisher matrix and MCMC analysis. As an example, we assume a typical BNS and BBH located at a representative redshift *z* = 0.84 and use the design sensitivity of DECIGO. The left panel shows the relative uncertainty $\Delta {{{{\mathcal{A}}}}}_{{{{\rm{mod}}}}}/{{{{\mathcal{A}}}}}_{{{{\rm{mod}}}}}$ as a function of the modulating SMBHB frequency ${f}_{{{{\rm{mod}}}}}$. The sensitivity curves have a characteristic shape that is similar to those of pulsar timing arrays^4,5^, with a peak sensitivity around ${f}_{{{{\rm{mod}}}}}\approx 1/T$. The drop in sensitivity below ${f}_{{{{\rm{mod}}}}}\lesssim 1/T$ reflects the fact that the observation time needs to cover at least one period of the SMBHB GW in order to establish its existence^6^. Conversely, the sensitivity degrades for higher frequencies following the ${f}_{{{{\rm{mod}}}}}T\gg 1$ limit of the hypergeometric functions in equation (19). The right panel of Fig. 2 shows the minimum detectable chirp mass ${{{{\mathcal{M}}}}}_{{{{\rm{mod}}}}}={({{{{\mathcal{A}}}}}_{{{{\rm{mod}}}}}d\,)}^{3/5}/{(\uppi {f}_{{{{\rm{mod}}}}})}^{2/5}$ of the modulating SMBHB. Masses as low as ${{{{\mathcal{M}}}}}_{{{{\rm{mod}}}}} \approx {{{\mathcal{O}}}}(1{0}^{7})\,{{{{\rm{M}}}}}_{\odot }$ can be detected if the compact binaries are located at a distance *d* = 1 pc to the SMBHB, as suggested by several binary formation channels (see below).

**Fig. 3:**
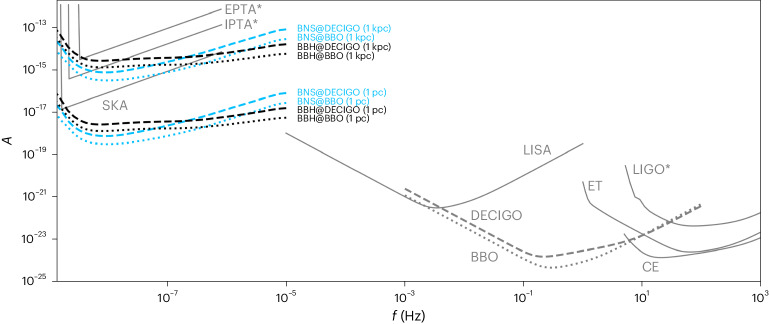
Landscape of different GW detectors compared to the method proposed in this work. For each detector, we plot the dimensionless strain amplitude ${{{\mathcal{A}}}}=\sqrt{f{S}_{n}}$ (solid lines) as function of frequency *f* (refs. ^2,3,101–106^), where the asterisks indicate currently operating detectors (in contrast to planned detectors). The black and blue lines show the equivalent strain sensitivity of our method, which corresponds to the GW strain amplitude the SMBHB would produce on Earth if it were indirectly detected (with $\Delta {{{{\mathcal{A}}}}}_{{{{\rm{mod}}}}}/{{{{\mathcal{A}}}}}_{{{{\rm{mod}}}}}=1{0}^{-1/2}$) through the modulations of a directly detected signal from a typical BBH (for example, GW150914 (ref. ^100^)) and a typical BNS (for example, GW170817 (ref. ^68^)), respectively. The dashed lines correspond to an observation with DECIGO and the dotted lines to an observation with BBO. We emphasize that this method can only detect SMBHBs that are concurrent with a compact object merger in its proximity at a given separation *d*, which limits the number of potential sources (see main text). Nevertheless, the resulting sensitivity can greatly outperform other nanohertz observatories, such as the European Pulsar Timing Array (EPTA), the International Pulsar Timing Array (IPTA) and SKA.

In Fig. 3, we compare the sensitivity of our method with other currently operating and planned GW detectors. Since the SMBHBs perturb compact binaries in their proximity, we consider an equivalent strain sensitivity that corresponds to the strain amplitude the distant SMBHBs would produce on Earth. The resulting equivalent sensitivities of DECIGO/BBO in the nanohertz band are comparable to or better than those of current and planned pulsar timing arrays. For distances between the stellar-mass compact binary and the SMBHB of *d* = 1 pc, suggested by various compact object binary formation channels (see below), the obtained sensitivity would outperform the anticipated sensitivity of the Square Kilometre Array (SKA) by two orders of magnitude. However, we stress that this striking sensitivity only applies to SMBHBs that are accompanied by a compact binary in their proximity, inherently limiting the number of detectable systems. The sensitivity differences between BNSs and BBHs are caused by their different masses and frequency evolution (Methods). They are negligible for the purpose of estimating the detection rate (see below). Thus, we will henceforth refer to both as generic compact binary sources.

Given the sensitivity curves above, we estimate the expected number and properties of SMBHBs that cause detectable modulations on the GWs from compact binaries observed with decihertz instruments. For this purpose, we adopt an SMBHB population model based on the Millennium Simulation^7^. We then distribute compact binaries within galaxies following the latter’s total stellar mass and evaluate the probability that they coincide with an SMBHB that produces a detectable modulation (Methods).

**Fig. 4:**
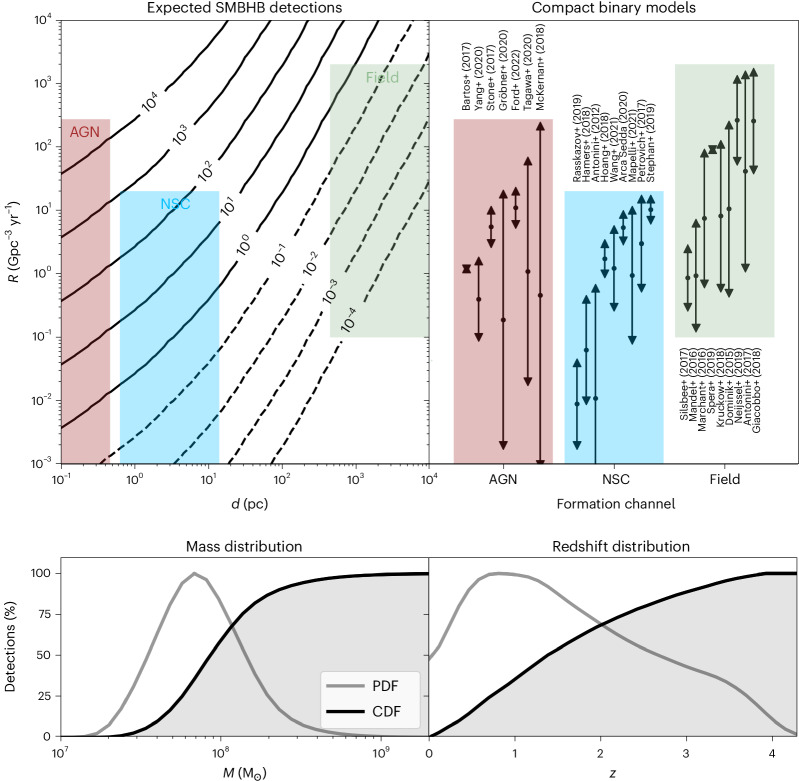
Total number and distribution of the expected SMBHB detections over a *T* = 10 years observation with a decihertz detector. Top: we show the expected number of SMBHB detections as a function of the compact binary merger rate ${{{\mathcal{R}}}}$ and the distance *d* from the Galactic centre at which the binaries typically merge. Compact binary mergers can occur through one or more formation channels. Rate predictions by several AGN^30–36^, NSC^20–29^ and field channel models^11–19^ are shown by coloured shaded areas and further detailed by the error bars in the top right. We assume a plausible range of $d\lesssim {{{\mathcal{O}}}}(1{0}^{-1})\,{{{\rm{pc}}}}, \approx {{{\mathcal{O}}}}(1{0}^{0})\,{{{\rm{pc}}}}$ and $\approx {{{\mathcal{O}}}}(1{0}^{3})\,{{{\rm{pc}}}}$ to represent the typical half-mass radii of AGN discs, NSCs and large galaxies, respectively. Crucially, the large majority of models for the AGN and NSC formation channels would guarantee tens to hundreds of SMBHB detections. Bottom: we show the mass and redshift distributions of the detectable SMBHBs in terms of their probability density (PDF) and cumulative density functions (CDF). The method is sensitive to individual sources with total mass *M* ≈ 10^7^–10^8^ M_⊙_ up to redshifts of *z* ≲ 3.5.

Figure 4 shows that the distribution of detectable SMBHBs strongly peaks within the range of 10^7^–10^9^ M_⊙_, which represents the best compromise between the total quantity of available SMBHBs and the strength of the modulation they produce. Most detections are limited to relatively low redshifts of 0.5 ≲ *z* ≲ 1. However, a substantial fraction of the potential detections is distributed in the large cosmological volume enclosed between *z* ≈ 1 and *z* ≈ 3.5. We highlight that for the majority of detected compact binaries the expected distance measurement and angular resolution of DECIGO/BBO would be sufficient to identify the host galaxy of the SMBHB^8^, allowing for targeted multi-messenger searches of subparsec SMBHBs. This shows how our method can unlock individual GW-based detections of the most massive SMBHBs in our Universe complementing current pulsar timing arrays and the upcoming LISA detector^9^ at higher redshifts and wider SMBHB separations, respectively.

Figure 4 also shows the expected total number of detectable SMBHBs as a function of the distance *d* and the compact binary merger rate ${{{\mathcal{R}}}}$. The distance *d* at which the compact binaries typically reside from the centre of their galaxy is a consequence of how it was formed; multiple formation channels have been proposed and are being actively discussed in literature to explain the origin of the compact binary mergers observed with LIGO-Virgo-Kagra^10^. For instance, in the field scenario compact binaries are formed far away from their galactic centre from the evolution of isolated massive stellar binaries, triples or more complex stellar systems^11–19^. In contrast, binary mergers near the galactic centre could be promoted by strong environmental effects in, for example, nuclear star clusters (NSCs)^20–29^ or inside the disks of active galactic nuclei (AGNs)^30–36^. While the latter have only been investigated for galactic centres that harbour a single supermassive black hole (SMBH), it is plausible to assume that they work similarly in galaxies that host SMBHBs. Given the current LIGO-Virgo-Kagra observations, it is uncertain which of these or other formation channels (for example, in globular clusters or young open star clusters) dominate the merger rate in the Universe, or whether multiple formation pathways co-exist^37^.

The majority of current NSC models yield a merger contribution of $$\begin{array}{l}{\mathcal{R}} \approx {\mathcal{O}}\left(1{0}^{-1}-1{0}^{1}\right)\,{\rm{Gpc}}^{-3}\,{\rm{yr}}^{-1}\end{array}$$, and NSCs are observed to have a typical half-mass radius of $\sim {{{\mathcal{O}}}}(1{0}^{0})\,{{{\rm{pc}}}}$. As shown in Fig. 4, this would guarantee ${N}_{\det } \approx {{{\mathcal{O}}}}(10)$ detections of SMBHBs after *T* = 10 years of observation. In the AGN channel, compact binaries merge even closer to the galactic centre, as they are within the extent $d\lesssim {{{\mathcal{O}}}}(1{0}^{-1})\,{{{\rm{pc}}}}$ of the AGN disk. Thus, we find that current AGN models allow for ${N}_{\det } \approx {\mathcal{O}}\left(1{0}^{-1}-10^4\right)$ SMBHB detections. Note that our method allows SMBHB detections even if these channels only amount to a subdominant fraction of the total rate of compact binary mergers^38^. Only in the least favourable scenario, in which all compact binaries are exclusively formed in the galactic field, do we expect no detections. In that case, we can assume the compact binaries to follow the galactic stellar mass distribution. Then, *d* is similar to the typical half-mass radius $\sim {{{\mathcal{O}}}}(1{0}^{3})\,{{{\rm{pc}}}}$ of massive galaxies, yielding ${N}_{\det }\lesssim {{{\mathcal{O}}}}(1{0}^{-1})$ for ${\mathcal{R}}\lesssim {\mathcal{O}}\left(10^{1}-10^{3}\right)\,{\rm{Gpc}}^{-3}\,{\rm{yr}}^{-1}$. Hence, it is plausible that a substantial number of compact binaries experience a detectable modulation due to the GWs from central SMBHBs, unless all of them merge in the galactic field.

Finally, our method can be used to distinguish different compact binary formation channels. For instance, the observation of a modulated compact binary would suggest a formation within a galactic nucleus, whereas the absence of such detection places strong upper limits of ${{{\mathcal{R}}}}\lesssim {{{\mathcal{O}}}}(1{0}^{-3})\,{{{{\rm{Gpc}}}}}^{-3}\,{{{{\rm{yr}}}}}^{-1}$ (AGN) and $\lesssim {{{\mathcal{O}}}}(1{0}^{-2})\,{{{{\rm{Gpc}}}}}^{-3}\,{{{{\rm{yr}}}}}^{-1}$ (NSC) on the rate of compact binary mergers that originate from the galactic centres.

It is important to note that we have restricted our analysis to sources that exhibit at least one entire sinusoidal modulation. In this way, we exclude the possibility that the perturbation to the GW may be degenerate with any additional slowly accumulating phase shift. For instance, GWs from a compact binary orbiting a central (single or binary) SMBH may exhibit a detectable phase shift caused by Doppler motion if $d\lesssim {{{\mathcal{O}}}}(1)\,{{{\rm{pc}}}}$ (ref. ^39^). This Doppler modulation may only be periodic, and thus degenerate with our effect, if the observational time is comparable or longer than the orbital period of the binary around the SMBH(B)s. For *T* = 10 years and *M* = 10^8^ M_⊙_, that is the case only if $d\lesssim {{{\mathcal{O}}}}(1{0}^{-2})\,{{{\rm{pc}}}}$. Such small separations arise exclusively in formation channels within migration traps of AGN disks^40,41^. Conversely, the simultaneous detection of our effect and the acceleration of the compact binary^42^ in the vicinity of a central SMBHBs would be an unambiguous signature of its existence. Furthermore, the periodicity of the sinusoidal modulation occurs on nanohertz frequencies. While strong gravity effects such as apsidal and spin precession may produce some oscillatory phase modulations, they occur on frequencies that are several orders of magnitude higher for binaries in the decihertz band. Additionally, the latter is evolving throughout the chirp and can therefore not be degenerate with a constant low frequency modulation (see also Supplementary Fig. 1, where we tested the degeneracy explicitly with the effective spin parameter).

To conclude, we have shown that the inherent sensitivity of proposed laser interferometers may be exploited to detect individual SMBHBs in the previously inaccessible mass range ≳10^7^ M_⊙_, at redshifts beyond the capacity of both current and future pulsar timing arrays. Currently, there are no other proposed methods that would realistically guarantee a direct detection of GWs from individual SMBHBs in the nanohertz band. A decihertz GW detector has exceptional scientific potential and should therefore be pursued with urgency, as it would open a large volume of the Universe to GW-based observations, simultaneously in both the decihertz and the nanohertz band.

## Methods

### Parameter estimation

A standard quantity to estimate the detectability of a GW strain *h* with a given detector is the SNR *ρ* defined as

3 $$\rho [h]=\sqrt{\left(h| h\right)},$$

where we defined the noise-weighted inner product

4 $$\left(a| b\right)=2\int_{{f}_{\min }}^{{f}_{\max }}{{{\rm{d}}}}f\,\frac{\tilde{a}(\;f\;){\tilde{b}}^{* }(\;f\;)+\tilde{b}(\;f\;){\tilde{a}}^{* }(\;f\;)}{{S}_{n}(\;f\;)}.$$

Here, *S*_*n*_(*f*) is the noise power spectral density of the detector and the integration boundaries ${f}_{\min }$ and ${f}_{\max }$ correspond to the signal frequencies at the beginning and the end of the observation, respectively. In general, the waveform *h* depends on a set of physical parameters *h* = *h*(*θ*^0^, *θ*^1^, *θ*^2^, …).

Let ${\hat{\theta }}^{i}$ be the correct values of the signal and ${\hat{\theta }}^{i}+\Delta {\theta }^{i}$ the best-fit parameters for a given realization of the noise. For a large SNR *ρ*, the measurement uncertainty Δ*θ*^*i*^ follows a Gaussian distribution^43–45^

5 $$p(\Delta {\theta }^{i})\propto \exp \left(-\frac{1}{2}{\varGamma }_{ij}\Delta {\theta }^{i}\Delta {\theta }^{\,j}\right),$$

where *Γ*_*ij*_ is the Fisher information matrix defined as

6 $${\varGamma }_{ij}=\left(\left.\frac{\partial h}{\partial {\theta }^{i}}\right\vert \frac{\partial h}{\partial {\theta }^{\,j}}\right).$$

Thus, the root-mean-square error of *θ*^*i*^ is

7 $$\sqrt{\left\langle {\left(\Delta {\theta }^{i}\right)}^{2}\right\rangle }=\sqrt{{\varSigma }^{ii}},$$

where *Σ* = *Γ*^−1^ is the inverse matrix of *Γ*. Thus, the relative uncertainty of the *i*th parameter can be estimated as $\sqrt{{\varSigma }^{ii}}/{\hat{\theta }}^{i}$.

The limitations of the Fisher matrix formalisms have been thoroughly explored in ref. ^45^. It is generally understood that, when it comes to GW parameter estimation, reliable estimates often require the full sampling of parameter posteriors to test for degeneracies and other features of the low-SNR regime. In GW parameter estimation, the goal is to maximize a likelihood ${{{\mathcal{L}}}}$ of the form

8 $${{{\mathcal{L}}}}\left(\varTheta \right)\propto \exp \left[-\left(h(\varTheta )-h({\varTheta }_{{{{\rm{inj}}}}})| h(\varTheta )-h({\varTheta }_{{{{\rm{inj}}}}})\right)\right],$$

where *Θ*_inj_ represent the parameters of an injected signal. In this work, we complement our Fisher matrix estimates with a series of MCMC-based parameter recovery tests. We then adjust the Fisher matrix detectability threshold to reflect the more conservative MCMC-based results. To explore the posterior distribution functions for the parameters *Θ*, we use the affine-invariant ensemble sampler emcee^46^ for MCMC proposed by ref. ^47^ and perform several tests with PN waveform models. In the MCMCs, we use uniform priors for all waveform parameters, except ${{{{\mathcal{A}}}}}_{{{{\rm{mod}}}}}$ and ${f}_{{{{\rm{mod}}}}}$ for which we impose log-uniform priors between the wide ranges 10^−12^–10^−4^ and 10^−12^–10^−4^ Hz, respectively. A realization of an MCMC test for a detectable modulation can be seen in Supplementary Fig. 1. In Supplementary Fig. 2, we show the sensitivity curve that results from injecting a grid of values of ${{{{\mathcal{A}}}}}_{{{{\rm{mod}}}}}$ and ${f}_{{{{\rm{mod}}}}}$. This sensitivity curve is used in Fig. 2.

### Unmodulated waveform

The simplest waveform from a circular binary can be obtained by modelling it as two Newtonian point masses *m*_1_ and *m*_2_ whose orbital frequency grows secularly due to the energy loss by GWs^44^:

9 $${\tilde{h}}_{{{{\rm{Newton}}}}}(\;f\;)=\frac{Q}{D}{{{{\mathcal{M}}}}}^{5/6}{f}^{\;-7/6}\exp [i\varPsi (\;f\;)],$$

where *f* ≥ 0, *D* is the luminosity distance to the binary, *Q* is a numerical factor accounting for the angular emission pattern of the source and the antenna response of the detector to the GW, and there is a phase

10 $$\varPsi (\;f\;)=2\uppi f{t}_{{\mathrm{c}}}-{\phi }_{{\mathrm{c}}}-\frac{\uppi }{4}+\frac{3}{4}{\left(8\uppi {{{\mathcal{M}}}}f\;\right)}^{-5/3}.$$

Thus, this waveform can be written in terms of four physical parameters: an overall amplitude ${{{\mathcal{A}}}}=Q{{{{\mathcal{M}}}}}^{5/6}/D$, a chirp mass ${{{\mathcal{M}}}}={({m}_{1}{m}_{2})}^{3/5}/{({m}_{1}+{m}_{2})}^{1/5}$, a collision time *t*_c_ describing the point in time of the merger, and a constant phase *ϕ*_c_. If the binary is at a cosmological distance at redshift *z*, we need to replace its chirp mass by ${{{\mathcal{M}}}}\to {{{\mathcal{M}}}}(1+z)$.

The advantage of Newtonian waveforms is that they may be easily treated in the Fisher matrix formalism and that the scalings and degeneracies are well understood^44^. However, Newtonian waveforms do not suffice for the requirements of parameter estimation already in current GW data analysis pipelines. To surpass these limitations, we include PN corrections for our Fisher matrix estimate and the MCMC-based numerical tests. We include the full phase evolution for non-spinning binaries up to 3 PN, and include the effect of spin through an additional phase modification at 1.5 PN order. The coefficients of the GW phase are taken from ref. ^48^. We also include the effect of all higher-order modes up to powers of *x*^2.5^, where *x* is the PN parameter, also taken from ref. ^48^. Effectively, this introduces also a dependency of the waveform on the reduced mass *μ* = *m*_1_*m*_2_/(*m*_1_ + *m*_2_) and the effective spin parameter *β*.

### Modulated waveform

We consider the situation where we have a ‘carrier’ source that emits GWs within the frequency bandwidth of our detector and another ‘background’ source radiating at much lower frequencies. The background GW modulates the apparent frequency of the carrier GW by perturbing spacetime along the detector–carrier line of sight. Thus, the measured frequency can be written as^49^

11 $${f}_{{{{\rm{measured}}}}}(t)=\frac{1}{T(t)+\Delta T(t)}\approx f(t)-f(t)\zeta (t)+{{{\mathcal{O}}}}({\zeta }^{\,2}),$$

where we substituted the unmodulated frequency *f*(*t*) ≡ 1/*T*(*t*) and a small modulation *ζ*(*t*) = Δ*T*(*t*)/*T*(*t*) ≪ 1. Note that the modulating effect of the background GW applies to any generic periodic carrier signal. For instance, using telescopes to search for modulations of the rotational period of millisecond pulsars is the aim of large-scale observational campaigns to detect low-frequency GWs^50–59^. In this work, we assume that the carrier source is a binary emitting GW (see above) whose frequency evolves as^60^

12 $$f(t)=\frac{1}{8\uppi }{\left[\frac{1}{5}({t}_{{\mathrm{c}}}-t){{{{\mathcal{M}}}}}^{5/3}\right]}^{-3/8}.$$

The modulation is given by^49^

13 $$\zeta (t)=\frac{{D}^{i}{D}^{j}}{2(1+\hat{{{{\bf{b}}}}}\cdot \hat{{{{\bf{D}}}}})}{h}_{ij}^{{{{\rm{TT}}}}}(t,{{{\bf{x}}}}=0)-\frac{{D}^{i}{D}^{j}}{2(1+\hat{{{{\bf{d}}}}}\cdot \hat{{{{\bf{D}}}}})}{h}_{ij}^{{{{\rm{TT}}}}}(t-D,{{{\bf{x}}}}={{{\bf{D}}}}),$$

where **x** = 0 is the position of the Earth, **x** = **D** the position of the carrier, **x** = **b** the position of the background source, **d** = **b** − **D** the vector from the background source to the carrier, and *h*^TT^ the linear spacetime metric perturbation in the transverse-traceless gauge that is induced by the background source. Equation (13) is identical to the formalism used to describe the GW-induced modulation of a pulsar^49^, except that in that case Earth and pulsar are considered much closer than the SMBHB (*D* ≪ *d*) so that the GW is incident from the same direction, that is, $\hat{{{{\bf{b}}}}}\approx \hat{{{{\bf{d}}}}}$.

In this work, we are considering the opposite case where the carrier and the background source are close to each other, but far away from Earth, that is, *D* ≫ *d* and $\hat{{{{\bf{b}}}}}\ne \hat{{{{\bf{d}}}}}$. In pulsar timing literature, the first term on the right-hand side (r.h.s.) of equation (13) is usually referred to as Earth term and the second to as pulsar term. The amplitudes of the first and second term on the r.h.s. of equation (13) decrease with distance *D* and *d*, respectively. Due to the large distance to the background source, we can neglect the former in our work and focus on the spacetime distortion the background source produces at the location of the carrier. If the background source is an SMBHB with a chirp mass ${{{{\mathcal{M}}}}}_{{{{\rm{mod}}}}}$ that emits monochromatic GWs at a frequency ${f}_{{{{\rm{mod}}}}}$, the distant modulation term can be written as^49,61^

14 $$\zeta (t)={{{{\mathcal{A}}}}}_{{{{\rm{mod}}}}}\cos (2\uppi {f}_{{{{\rm{mod}}}}}t+{\phi }_{{{{\rm{mod}}}}}),$$

where its amplitude is of the order ${{{{\mathcal{A}}}}}_{{{{\rm{mod}}}}}\approx {(\uppi {f}_{{{{\rm{mod}}}}})}^{2/3}{{{{\mathcal{M}}}}}_{{{{\rm{mod}}}}}^{5/3}/d$. Here, we averaged over a random orientation of **d** with respect to **D** and a random inclination of the orbital plane of the SMBHB. In the time domain, the GW strain from the carrier is given by^44^

15 $$h(t)=\frac{{(384/5)}^{1/2}{\uppi }^{2/3}Q\mu M}{Dr(t)}\cos \left(2\uppi {\int}^{t}{f}_{{{{\rm{measured}}}}}({t}^{{\prime} })\,{{{\rm{d}}}}{t}^{{\prime} }\right),$$

where *M* = *m*_1_ + *m*_2_ and *μ* = *m*_1_*m*_2_/*M* are the total and reduced mass, respectively, and the orbital separation of the binary is

16 $$r(t)={\left(\frac{256}{5}\mu {M}^{2}\right)}^{1/4}{\left({t}_{{\mathrm{c}}}-t\right)}^{1/4}.$$

Considering the integral in equation (15), we have

17 $$\begin{array}{rc}\phi (t)&\equiv 2\uppi {\displaystyle\int}^{t}\;{f}_{{{{\rm{measured}}}}}({t}^{{\prime} })\,{{{\rm{d}}}}{t}^{{\prime} }\approx 2\uppi {\displaystyle\int}^{t}\;f({t}^{{\prime} })\,{{{\rm{d}}}}{t}^{{\prime} }-2\uppi {\displaystyle\int}^{t}f({t}^{{\prime} })\zeta ({t}^{{\prime} })\,{{{\rm{d}}}}{t}^{{\prime} },\end{array}$$

where the first integral on the r.h.s. evaluates to^44^

18 $$2\uppi {\int}^{t}f({t}^{{\prime} })\,{{{\rm{d}}}}{t}^{{\prime} }=-2{\left(\frac{T}{5{{{\mathcal{M}}}}}\right)}^{5/8}+{\phi }_{{\mathrm{c}}},$$

where *T* = *t*_c_ − *t* is the time to coalescence and the second integral becomes

19 $$\begin{array}{l}2\uppi {\displaystyle\int}^{t}f({t}^{{\prime} })\zeta ({t}^{{\prime} })\,{{{\rm{d}}}}{t}^{{\prime} }=-\frac{2\,{{{{\mathcal{A}}}}}_{{{{\rm{mod}}}}}T}{13\times {5}^{5/8}{\left(T{{{{\mathcal{M}}}}}^{5/3}\right)}^{3/8}}\\ \times \left[10\uppi\; {f}_{{{{\rm{mod}}}}}T\sin {({\phi }_{{{{\rm{mod}}}}})}_{1}{F}_{2}\left(\frac{13}{16};\frac{3}{2},\frac{29}{16};-{f}_{{{{\rm{mod}}}}}^{\;2}{T}^{\;2}{\uppi }^{2}\right)\right.\\ \left.+13\cos {({\phi }_{{{{\rm{mod}}}}})}_{1}{F}_{2}\left(\frac{5}{16};\frac{1}{2},\frac{21}{16};-{f}_{{{{\rm{mod}}}}}^{\;2}{T}^{\;2}{\uppi }^{2}\right)\right]+{\phi }_{{\mathrm{c}},{{{\rm{mod}}}}}.\end{array}$$

The hypergeometric functions _1_*F*_2_ evaluate to one in the limit $(\;{f}_{{{{\rm{mod}}}}}T\;)\to 0$ where the period of the modulating GW is much longer than the observation time, and they are zero in the opposite regime $(\;{f}_{{{{\rm{mod}}}}}T\;)\to \infty$. Next, we consider the Fourier transform

20 $$\tilde{h}(\;f\;)=\int_{-\infty }^{\infty }h(t){{\mathrm{e}}}^{2\uppi ift}\,{{{\rm{d}}}}t.$$

In the stationary-phase approximation, we get^44^

21 $$\tilde{h}(\;f\;)=\frac{Q}{D}{{{{\mathcal{M}}}}}^{5/6}{f}^{\;-7/6}\exp \left[i(2\uppi ft-\phi (\;f\;)-\uppi /4)\right],$$

where *ϕ*(*f*) ≡ *ϕ*(*f*(*T*)) and

22 $$\begin{array}{rc}T(\;f\;)&=5{(8\uppi f\;)}^{-8/3}{{{{\mathcal{M}}}}}^{-5/3}.\end{array}$$

Note that equation (21) reduces to equation (9) if there is no modulation ${{{{\mathcal{A}}}}}_{{{{\rm{mod}}}}}=0$. In general, the modulated waveform in equation (21) now depends on four additional physical parameters: an overall amplitude ${{{{\mathcal{A}}}}}_{{{{\rm{mod}}}}}$, a modulating frequency ${f}_{{{{\rm{mod}}}}}$ and the phases ${\phi }_{{{{\rm{mod}}}}}$ and ${\phi }_{{{{\rm{c}}}},{{{\rm{mod}}}}}$. However, we can further reduce the degrees of freedom to seven in total by omitting ${\phi }_{{{{\rm{c}}}},{{{\rm{mod}}}}}$ through an appropriate redefinition of ${\phi }_{{\mathrm{c}}}\to {\phi }_{{\mathrm{c}}}+{\phi }_{{\mathrm{c}},{{{\rm{mod}}}}}$. When we calculate the noise-weighted inner product in equation (4), the integration limits are chosen to be ${f}_{\min }=f(T=10\,{{{\rm{years}}}})$ and ${f}_{\max }={f}_{{{{\rm{ISCO}}}}}\equiv 1/12\uppi \sqrt{6}({m}_{1}+{m}_{2})$, that is, we assume that we observe all binaries during their last 10 years before they merge. In reality, when a detector begins to observe the binaries will be distributed uniformly in *T*, that is, some of the binaries are in fact observed for less than 10 years before they merge (${f}_{\min } > f(T=10\,{{{\rm{years}}}})$). Binaries that are observed for less than 10 years are less likely to exhibit one full cycle of a low-frequency modulation, meaning that the modulation is more liable to be degenerate with other environmental effects (see main text). However, for the typical carrier sources we are considering, more than ~90 % of the SNR is accumulated in the last year before merger. Indeed, we find that the bound on $\Delta {{{{\mathcal{A}}}}}_{{{{\rm{mod}}}}}/{{{{\mathcal{A}}}}}_{{{{\rm{mod}}}}}$ converges to the results shown in Fig. 2 well within a factor of 2 for sources that are observed for ~3 years or more. Thus, our population estimates will at most be reduced by ~30 % by this consideration.

### Carrier populations

During the first three LIGO-Virgo-Kagra observing runs, the GWs of ninety mergers of BBHs, BNSs and neutron star–black hole (NSBH) binaries have been directly detected^38,62–65^. From this sample, it is possible to reconstruct the merger rate and parameter distribution of the underlying astrophysical population of compact binaries^38^. Owing to their stronger signal, most mergers have been detected of BBHs. Their inferred merger rate in the local Universe is^38^

23 $${{{{\mathcal{R}}}}}_{{{{\rm{BBH}}}}}=17.9\,\,-\,\,44\,{{{{\rm{Gpc}}}}}^{-3}\,{{{{\rm{yr}}}}}^{-1}\quad (90 \% \,{{{\rm{CI}}}}),$$

at a fiducial redshift *z* = 0.2. The rate is observed to scale with redshift as (1 + *z*)^*κ*^, where $\kappa =2.{9}_{-1.8}^{+1.7}$ for *z* ≲ 1. It is plausible to assume that this trend extends to redshifts *z* ≈ 2–3 if it roughly follows the shape of the cosmic star formation rate density of massive stars^66,67^. In addition, the detection catalogue of the first three LIGO-Virgo-Kagra observing runs contains two BNSs and four NSBH binaries^38^. The inferred merger rates in the local Universe are

24 $${{{{\mathcal{R}}}}}_{{{{\rm{BNS}}}}}=10\,\,-\,\,1700\,{{{{\rm{Gpc}}}}}^{-3}\,{{{{\rm{yr}}}}}^{-1}\quad (90 \% \,{{{\rm{CI}}}}),$$

25 $${{{{\mathcal{R}}}}}_{{{{\rm{NSBH}}}}}=7.8\,\,-\,\,140\,{{{{\rm{Gpc}}}}}^{-3}\,{{{{\rm{yr}}}}}^{-1}\quad (90 \% \,{{{\rm{CI}}}}).$$

Due to the small number of detections these rate estimates are quite uncertain. Furthermore, no redshift evolution could be determined in either cases. For the purpose of this work, we agnostically assume the same redshift scaling as for the BBH mergers.

Regarding the chirp mass of the mergers, the BBHs follow a clumped distribution with pronounced overdensities around ${{{\mathcal{M}}}}=8.{3}_{-0.5}^{+0.3}\,{{{{\rm{M}}}}}_{\odot }$ and $27.{9}_{-1.8}^{+1.9}\,{{{{\rm{M}}}}}_{\odot }$. Meanwhile, the chirp masses of the six events involving a neutron star range from ${{{\mathcal{M}}}}=1.18{8}_{-0.002}^{+0.004}\,{{{{\rm{M}}}}}_{\odot }$ (GW170817 (ref. ^68^)) to 3.7 ± 0.2 M_⊙_ (GW190917 (ref. ^69^)). The chirp mass of the carriers affects our results in two ways. On the one hand, a higher chirp mass increases the SNR of the carrier, which overall benefits the identification of a modulating background source. On the other hand, a higher chirp mass shortens the merger time and the time a binary spends in the frequency bandwidth of a detector (cf., equation (22)). This impedes the identification of a modulating background source in a more important way. To demonstrate this effect, we adopt ${{{\mathcal{M}}}}=27.9\,{{{{\rm{M}}}}}_{\odot }$ from the first directly detected BBH merger (GW150914 (ref. ^70^)) as a fiducial value for the chirp mass of a rather massive equal-mass BBH and assume the chirp mass ${{{\mathcal{M}}}}=1.188\,{{{{\rm{M}}}}}_{\odot }$ of an equal-mass BNS as our standard choice for binaries with a neutron star component.

### Proposed decihertz detectors

A GW detector that is sensitive to frequencies around $\gtrsim {{{\mathcal{O}}}}(1{0}^{-2})\,{{{\rm{Hz}}}}$ is ideal for our type of analysis. In this frequency range, the compact binaries that we consider in this work spend a time $T\lesssim f/\dot{f}$ up to several years before they merge in the bandwidth of LIGO-Virgo-Kagra. Monitoring carrier binaries over several years allows us to detect frequencies of a modulating background source as low as ${f}_{{{{\rm{mod}}}}}\gtrsim 1/T \sim {{{\mathcal{O}}}}(1{0}^{-9})\,{{{\rm{Hz}}}}$, which would be a probe of the most massive black-hole binaries in the Universe. For this reason, we focus on existing detector proposals in the decihertz regime. For concreteness, we consider the space-based instruments DECIGO^71,72^ and BBO^73–75^ but stress that our analysis could be applied to any detector that is sensitive to those frequencies. We note that DECIGO is actively being developed, while we are not aware of any research efforts towards the realization of BBO.

Both DECIGO and BBO consist of multiple (up to four) triangular constellations of spacecraft in heliocentric orbits, where the constellations are equally spaced around the ecliptic. DECIGO and BBO are designed to detect GWs at frequencies between 0.1 Hz and 10 Hz such that they could provide sensitivity in a frequency range between the sensitive band of LISA and ground-based detectors such as LIGO-Virgo-Kagra. Both DECIGO and BBO are laser-interferometric detectors where the interferometer arms are rendered by beams of laser light that travel between the spacecraft. In general, the sensitivity of interferometers decreases above a frequency inversely proportional to the arm length, and therefore both DECIGO and BBO will employ arms shorter than those of LISA. DECIGO plans to use interferometer arm lengths ~10^3^ km; BBO’s arms are intended to have a length of ~5 × 10^4^ km (cf. LISA, which has a design with arm length ~2.5 × 10^6^ km)^9,74,76^. While BBO has arms that are longer than those of DECIGO by a factor of ~10, DECIGO is designed with arms that contain Fabry–Perot cavities with a finesse of ~10, and the instruments therefore have almost equal strain sensitivities. The optical frequency response of both instruments is comparable as well: the sensitivity to GWs falls off linearly at frequencies above the pole frequency of the Fabry-Perot cavities in the arms of DECIGO, and similary above the light-crossing frequency of the arms of BBO. The design of DECIGO envisions the instrument to be limited by quantum noise across the entire band, specifically quantum radiation pressure noise and quantum shot noise. Extensive mitigation of classical low-frequency acceleration noise will be required to achieve this condition. Quantum radiation pressure noise falls off as *f*^−2^, and quantum shot noise is constant at all frequencies; the crossover point of these two noise contributions is determined by the optical power circulating in the arms. BBO is expected to be limited at low frequency by acceleration noise of the test masses due to a variety of sources, and at high frequencies by quantum shot noise.

For both BBO and DECIGO, the triangular design of the instrument provides two independent interferometric measurements of incident GWs, which can be equivalently described as measurements from two independent L-shaped Michelson interferometers^2,3,9,77^. Given the proposed experimental parameters, the quantum noise and optical response may be modelled, and the sensitivity of of each independent L-shaped interferometer can thus be described as an effective noise power spectral density *S*_n_. Here, we use the analytical expressions^2,3^ for DECIGO

26 $$\begin{array}{rcl}{S}_{{\mathrm{n}}}^{{{{\rm{DECIGO}}}}}(\;f\;)&=&7.05\times 1{0}^{-48}\left[1+{\left(\frac{f}{{f}_{{\mathrm{p}}}}\right)}^{2}\right]{{{{\rm{Hz}}}}}^{-1}\\ &&+4.8\times 1{0}^{-51}{\left(\frac{f}{1\,{{{\rm{Hz}}}}}\right)}^{-4}\frac{1}{1+{\left(\frac{f}{{f}_{{\mathrm{p}}}}\right)}^{2}}\,{{{{\rm{Hz}}}}}^{-1}\\ &&+5.33\times 1{0}^{-52}{\left(\frac{f}{1\,{{{\rm{Hz}}}}}\right)}^{-4}\,{{{{\rm{Hz}}}}}^{-1},\end{array}$$

where *f*_p_ = 7.35 Hz and for BBO

27 $$\begin{array}{rcl}{S}_{{\mathrm{n}}}^{{{{\rm{BBO}}}}}(\;f\;)&=&2.00\times 1{0}^{-49}{\left(\frac{f}{1\,{{{\rm{Hz}}}}}\right)}^{2}\,{{{{\rm{Hz}}}}}^{-1}\\ &&+4.58\times 1{0}^{-49}\,{{{{\rm{Hz}}}}}^{-1}\\ &&+1.26\times 1{0}^{-51}{\left(\frac{f}{1\,{{{\rm{Hz}}}}}\right)}^{-4}\,{{{{\rm{Hz}}}}}^{-1}.\end{array}$$

The sensitivity further depends on the implementation of the instruments; the achievable SNR increases as $\sqrt{{N}_{{{{\rm{IFO}}}}}}$, where *N*_IFO_ is the number of independent L-shaped Michelson interferometers that the detector furnishes. In this paper, for both DECIGO and BBO, we take a sensitivity corresponding to one triangular constellation, such that *N*_IFO_ = 2, that is, we divide equations (26) and (27) by $\sqrt{2}$. Furthermore, we assume a nominal mission lifetime for both instruments of *T* = 10 years. In assessing the sensitvity of DECIGO/BBO, we also consider the angular response factor *Q* (cf. equation (9)). Averaging over the antenna pattern for sky positions (*ϑ*, *φ*) of an L-shaped Michelson and all possible binary orbital inclinations (*ι*) gives^61^

28 $$\sqrt{\langle | Q(\vartheta ,\varphi ;\iota ){| }^{2}\rangle }=\frac{2}{5},$$

which we will use for *Q* in this work.

### Background source population

The background sources considered in this work consist in individual SMBHBs, which can span over several decades in mass, mass ratio and orbital separation. The SMBHB merger rate is highly uncertain, and constraining it is one of the major scientific goals of proposed GW observatories such as LISA^9,76^, TianQin^78^, pulsar timing arrays^79–83^ and other electromagnetic searches^84,85^. For the purposes of this work, we broadly follow the procedure detailed in ref. ^86^ and link the SMBHB merger rate to the well-established halo merger rate, based on the two Millenium simulations^87^:

29 $$\frac{{{{{\rm{d}}}}}^{2}H}{{{{\rm{d}}}}\xi {{{\rm{d}}}}z}={B}_{1}{\left(\frac{{M}_{{{{\rm{halo}}}}}}{1{0}^{12}{{{{\rm{M}}}}}_{\odot }}\right)}^{{b}_{1}}{\xi }^{{b}_{2}}\exp \left[{\left(\frac{\xi }{{B}_{2}}\right)}^{{b}_{3}}\right]{(1+z)}^{{b}_{4}},$$

where *H* is the total number of mergers that a halo of mass *M*_halo_ experiences over cosmic time, *ξ* ≤ 1 is the halo merger mass ratio and the best-fit parameters are given by [*B*_1_, *B*_2_, *b*_1_, *b*_2_, *b*_3_, *b*_4_] = [0.0104, 9.72 × 9.72, 0.133, − 1.995, 0.263, 0.0993]. The SMBHB merger rate ${\dot{N}}_{\bullet \bullet }$ can be obtained by multiplying the halo merger rate with the SMBH mass function

30 $$\frac{{{{{\rm{d}}}}}^{3}{\dot{N}}_{\bullet \bullet }}{{{{\rm{d}}}}{M}_{\bullet }{{{\rm{d}}}}\xi {{{\rm{d}}}}z}=P({M}_{{{{\rm{halo}}}}},z)\frac{4\uppi {D}_{{{{\rm{com}}}}}^{2}(z)}{{(1+z)}^{3}}\frac{{{{\rm{d}}}}{n}_{\bullet }}{{{{\rm{d}}}}{M}_{\bullet }}\frac{{{{{\rm{d}}}}}^{2}H}{{{{\rm{d}}}}\xi {{{\rm{d}}}}z}(\xi ,{z}_{{{{\rm{del}}}}}),$$

where *c* = 1 and where we must supply an occupation fraction *P*, a relation between SMBH and halo masses, and a delay prescription between the nominal halo merger and the actual SMBHB merger. Considering that the most easily detectable modulations will be naturally produced by heavy, low-redshift binaries we can tailor equation (30) to SMBH masses of *M*_•_ > 10^8^ M_⊙_ within moderately low redshifts *z* < 4. First, we adopt the SMBH mass function reported in ref. ^88^, which we denote as

31 $$\phi \equiv \frac{{{{\rm{d}}}}{n}_{\bullet }}{{{{\rm{d}}}}\,{\log }_{10}{M}_{\bullet }},$$

where *ϕ* is the symbol customarily used to denote the SMBH mass function in units of Mpc^−3^ yr^−1^ dex^−1^. Secondly, we adopt a simple relation between halo and SMBH mass from ref. ^89^:

32 $${M}_{\bullet }={\left[\frac{{M}_{{{{\rm{halo}}}}}(1+z)}{2\times 1{0}^{7}\,{{{{\rm{M}}}}}_{\odot }}\right]}^{3/2}\,{{{{\rm{M}}}}}_{\odot },$$

which is broadly consistent with both theoretical and observational constraints^90,91^. Given the selection effects towards higher masses, we can safely assume an occupation fraction *P* ≈ 1 as it is strongly suggested by simulations^92^ and observations of AGN^93^. Finally, the time delays between the halo merger and the eventual SMBHB merger are expected to be of the order 10^8^–10^9^ years for the SMBHBs we are considering^94,95^. As seen in Supplementary Fig. 2, we have tested the effect of introducing constant time delays of up to 1 Gyr by shifting the merger redshift to *z* → *z* − Δ*z*(*τ*), where *τ* is the time delay and we assume a standard cosmology. We find that our results do not change appreciably, decreasing by at most ~20% and only at redshifts higher than ~3. This result runs counter to the common expectation that introducing delays reduces the number of detected massive BH mergers for GW detectors^96,97^. In our work, it can be attributed to two factors. Firstly, the halo merger rate reported in the Millennium simulation only weakly depends on redshift, scaling as ~(1 + *z*)^0.1^. Secondly, in our setup, shifting SMBHB mergers to lower redshift automatically increases the SNR of the detected stellar-mass compact object binaries while simultaneously reducing their cosmological redshift. This has the consequence to greatly expand the range of detectable modulations in both frequency and amplitude, counteracting the diminished number of SMBHB mergers. The fact that these effects seem to cancel out each other is somewhat of a coincidence, which we attribute to the explicit form of equation (29).

### Rate estimate

Given a population of both carriers and background sources, we can estimate the mean number of compact binaries that could exhibit a detectable modulation. To achieve this, we must select all SMBHBs that are emitting loud GWs in the appropriate frequency range for the sensitivity curves detailed above (cf. Fig. 2). The first step is to distribute the SMBHB over several frequency bins, by replacing the time derivative with an integration over ${f}_{{{{\rm{mod}}}}}$:

33 $${N}_{\bullet \bullet }=\int{\dot{N}}_{\bullet \bullet }{{{\rm{d}}}}t=\frac{{{{\rm{d}}}}{N}_{\bullet \bullet }}{{{{\rm{d}}}}{f}_{{{{\rm{mod}}}}}}{\dot{f}}_{{{{\rm{mod}}}}}{{{\rm{d}}}}t=\int\frac{{{{\rm{d}}}}{N}_{\bullet \bullet }}{{{{\rm{d}}}}{f}_{{{{\rm{mod}}}}}}\,{{{\rm{d}}}}{f}_{{{{\rm{mod}}}}}$$

Note that, since we are considering SMBHBs in the GW-driven regime, we can simply use equation (12) to describe the frequency evolution. Then, we can integrate the differential contributions over frequency and mass ratio, using a Heaviside function *Θ* to only select SMBHB that would produce detectable modulations, as defined by a certain confidence threshold ${\sigma }_{\det }$:

34 $$\frac{{{{{\rm{d}}}}}^{2}{N}_{\bullet \bullet }^{\,{{{\rm{mod}}}}}}{{{{\rm{d}}}}{M}_{\bullet }{{{\rm{d}}}}z}=\int\int\frac{{{{{\rm{d}}}}}^{4}{N}_{\bullet \bullet }}{{{{\rm{d}}}}{M}_{\bullet }{{{\rm{d}}}}\xi {{{\rm{d}}}}{f}_{{{{\rm{mod}}}}}{{{\rm{d}}}}z}\varTheta \left({\sigma }_{\det }-\frac{\Delta\, {{{{\mathcal{A}}}}}_{{{{\rm{mod}}}}}}{{{{{\mathcal{A}}}}}_{{{{\rm{mod}}}}}}\right)\,{{{\rm{d}}}}{f}_{{{{\rm{mod}}}}}\,{{{\rm{d}}}}\xi .$$

The Heaviside function depends on the sensitivity curves detailed above (cf. Fig. 2):

35 $$\varTheta =\varTheta (z,{{{{\mathcal{A}}}}}_{{{{\rm{mod}}}}},{f}_{{{{\rm{mod}}}}},{{{\mathcal{M}}}},{t}_{{\mathrm{c}}},{\phi }_{{\mathrm{c}}},{\phi }_{{{{\rm{mod}}}}}),$$

where we initially choose a threshold of $\Delta {{{{\mathcal{A}}}}}_{{{{\rm{mod}}}}}={{{{\mathcal{A}}}}}_{{{{\rm{mod}}}}}$, that is, ${\sigma }_{\det }=1$, to represent a SMBHB modulation, the parameters of which can be well constrained. Note that, typically, constraining the parameters of a GW to 1*σ* does not constitute a sufficient threshold to avoid the possibility of false signals. In our case however, we are considering modulations that affect GWs signals with SNR of order few 10^2^, and it is not immediately clear whether the same intuition applies, nor if the posterior distributions of the parameters are Gaussian. To test this, we have run additional MCMC tests in which the null hypothesis, that is, the absence of a signal, is tested against several realizations of Gaussian noise. We observe that the MCMC walkers freely sample regions in which the trial amplitude is very low. However, they are strongly discouraged to sample large values of ${{{{\mathcal{A}}}}}_{{{{\rm{mod}}}}}$, which are inconsistent with the absence of a signal. The posterior probability for the recovered modulation amplitude ${{{{\mathcal{A}}}}}_{{{{\rm{mod}}}}}$ sharply decreases above a certain threshold ${{{{\mathcal{A}}}}}_{{{{\rm{NH}}}}}$ associated to the null hypothesis

36 $${{{\mathcal{P}}}}({{{{\mathcal{A}}}}}_{{{{\rm{mod}}}}}) \sim \left\{\begin{array}{ll}\,{{\mbox{const}}}\,.\quad &\,{{\mbox{for}}}\,\,{{{{\mathcal{A}}}}}_{{{{\rm{mod}}}}} < {{{{\mathcal{A}}}}}_{{{{\rm{NH}}}}}(\;{f}_{{{{\rm{mod}}}}})\\ 0\quad &\,{{\mbox{for}}}\,\,{{{{\mathcal{A}}}}}_{{{{\rm{mod}}}}} > {{{{\mathcal{A}}}}}_{{{{\rm{NH}}}}}(\;{f}_{{{{\rm{mod}}}}}).\end{array}\right.$$

More precisely, the boundary is a half-Gaussian, for which the inflection point defines the 1*σ*_NH_ confidence threshold between the allowed and disallowed regions. We find that the boundary at which the null hypothesis can be confidently excluded always lies about one order of magnitude below our choice of sensitivity curve, especially at lower modulation frequencies, where the majority of detections take place (Supplementary Fig. 2). In this sense, our choice of using a 1*σ* boundary simply refers to the desired accuracy in the recovered modulation parameters, though it is likely that a more complex noise model would affect this conclusion. Therefore, the 1*σ* detection criterion of ${{{{\mathcal{A}}}}}_{{{{\rm{mod}}}}}$ only defines how accurate we could determine its value when a modulation is present and does not imply a large false positive probability at which we would wrongfully conclude the existence of a modulation. We have additionally tested how the number of detection scales as a function of the desired posterior widths, finding that

37 $${N}_{\det }({\sigma }_{\det })\propto {\sigma }_{\det }^{-1.2}.$$

This means that a substantial fraction of the detections will result in better-constrained posteriors.

The next step is to distribute the total amount of compact object mergers into their host galaxies and across cosmic time. To enable this calculation, we require to know the differential compact object binary rate per halo. The most natural assumption is that the latter must be proportional to the stellar mass of the galaxy that occupies a halo of a given size. Interestingly, for the high-mass galaxies likely to host heavy SMBHs, the stellar mass–halo mass relation has a turnover point at a value of ~1/100, varying by approximately one order of magnitude over the large range of 10^11^ M_⊙_ < *M*_halo_ < 10^14^ M_⊙_. For the purposes of this work, we use a fit of the stellar mass to halo mass ratio ${{{{\mathcal{F}}}}}^{\star }$ (refs. ^98,99^):

38 $${{{{\mathcal{F}}}}}^{\star }({M}_{{{{\rm{halo}}}}})=2{D}_{1}{(1+z)}^{{\delta }_{1}}{\left[{\left(\frac{{M}_{{{{\rm{Halo}}}}}}{{D}_{2}}\right)}^{-\beta }+{\left(\frac{{M}_{{{{\rm{Halo}}}}}}{{D}_{2}}\right)}^{\gamma }\right]}^{-1},$$

39 $${D}_{2}=1{0}^{{\delta }_{2}+z{\delta }_{3}}\,{{{{\rm{M}}}}}_{\odot },$$

40 $$\beta =z{\delta }_{4}+{\delta }_{5},$$

41 $$\gamma ={\delta }_{6}{(1+z)}^{{\delta }_{7}},$$

where the best-fit parameters are (*D*_1_, *δ*_1_, *δ*_2_, *δ*_3_, *δ*_4_, *δ*_5_, *δ*_6_, *δ*_7_) = (0.046, −0.38, 11.79, 0.20, 0.043, 0.96, 0.709, −0.18). Thus, the number of compact object binaries is distributed as

42 $$\frac{{{{\rm{d}}}}{{{{\mathcal{R}}}}}_{{{{\rm{COM}}}}}}{{{{\rm{d}}}}{M}_{{{{\rm{halo}}}}}}=\frac{{{{{\mathcal{R}}}}}_{{{{\rm{COM}}}}}}{{{{\mathcal{N}}}}}{{{{\mathcal{F}}}}}^{\star }({M}_{{{{\rm{halo}}}}}){M}_{{{{\rm{halo}}}}}\frac{{{{\rm{d}}}}{n}_{{{{\rm{halo}}}}}}{{{{\rm{d}}}}{M}_{{{{\rm{halo}}}}}},$$

where the normalization is given by an integral over the total stellar mass contained in all halos in the Universe:

43 $${{{\mathcal{N}}}}=\int{{{{\mathcal{F}}}}}^{\star }({M}_{{{{\rm{halo}}}}}){M}_{{{{\rm{halo}}}}}\frac{{{{\rm{d}}}}{n}_{{{{\rm{halo}}}}}}{{{{\rm{d}}}}{M}_{{{{\rm{halo}}}}}}\,{{{\rm{d}}}}{M}_{{{{\rm{halo}}}}},$$

and we obtain the halo mass function by inverting equation (32) and adjusting the differentials accordingly. Note that relaxing the assumption of a constant occupation fraction does not greatly affect the normalization because a large fraction of the total mass budget is composed by massive halos above ~10^12^ M_⊙_.

To estimate the probability of a compact object merger taking place in a galaxy that simultaneously hosts a modulating SMBHB, we suppress the former’s rate by a further factor representing the number of halos that contain such an SMBHB with respect to the total number of halos, in a given redshift shell with volume d*V*_*z*_:

44 $$\frac{{{{{\rm{d}}}}}^{2}{{{{\mathcal{R}}}}}_{{{{\rm{COM}}}}}^{{{{\rm{mod}}}}}}{{{{\rm{d}}}}{M}_{{{{\rm{halo}}}}}{{{\rm{d}}}}z}=\frac{{{{{\rm{d}}}}}^{2}{{{{\mathcal{R}}}}}_{{{{\rm{COM}}}}}}{{{{\rm{d}}}}{M}_{{{{\rm{halo}}}}}{{{\rm{d}}}}z}\frac{{{{{\rm{d}}}}}^{2}{N}_{\bullet \bullet }^{\,{{{\rm{mod}}}}}}{{{{\rm{d}}}}{M}_{{{{\rm{halo}}}}}{{{\rm{d}}}}z}{\left(\frac{{{{\rm{d}}}}{n}_{{{{\rm{halo}}}}}}{{{{\rm{d}}}}{M}_{{{{\rm{halo}}}}}}\frac{{{{\rm{d}}}}{V}_{z}}{{{{\rm{d}}}}z}\right)}^{-1},$$

where once again we make use of the halo mass–SMBH mass relation and adjust the differentials accordingly. Note that here the compact object merger rate must be transformed from the local source frame to the observer frame, by dividing the observed redshift dependence by a factor (1 + *z*) and expliciting the differential d/d*z*.

Finally, the total number of compact object mergers with a detectable modulation ${N}_{{{{\rm{COM}}}}}^{\,{{{\rm{mod}}}}}$ can be obtained by integrating and multiplying by the total observation time:

45 $${N}_{{{{\rm{COM}}}}}^{\,{{{\rm{mod}}}}}=T\int\frac{{{{{\rm{d}}}}}^{2}{{{{\mathcal{R}}}}}^{{{{\rm{mod}}}}}}{{{{\rm{d}}}}{M}_{{{{\rm{halo}}}}}{{{\rm{d}}}}z}{{{\rm{d}}}}{M}_{{{{\rm{halo}}}}}\,{{{\rm{d}}}}z\equiv {N}_{\det },$$

where, equivalently, ${N}_{\det }$ is the number of indirectly detected SMBHB. Note that in reality the number ${N}_{{{{\rm{COM}}}}}^{\,{{{\rm{mod}}}}}$ is actually the expectation value of a process that involves sampling both compact object binaries and SMBHB from a large pool of halos, analogous to a binomial distribution, excluding the unlikely possibility of having two separate compact object binaries merging simultaneously with an SMBHB in the same galaxy. Therefore, it carries an intrinsic uncertainty approximately proportional to its square root.

## Supplementary information

Supplementary Information Supplementary Figs. 1 and 2.

## Data Availability

The data and codes for reproducing the results of this work are available via GitHub at https://github.com/stegmaja/GW-GW-Modulations.
