# Supplementary material for: Imprints of massive black-hole binaries on neighbouring decihertz gravitational-wave sources
Source: Nat Astron. 2024 Aug 5;8(10):1321–31. doi: 10.1038/s41550-024-02338-0 (PMC11486661; doi:10.1038/s41550-024-02338-0)
Supplement: Supplementary file 1 — Supplementary Figs. 1 and 2. [file 41550_2024_2338_MOESM1_ESM.pdf]

nature astronomy

Supplementary information

https://doi.org/10.1038/s41550-024-02338-0

Imprints of massive black-hole binaries on neighbouring decihertz gravitational-wave sources

In the format provided by the authors and unedited

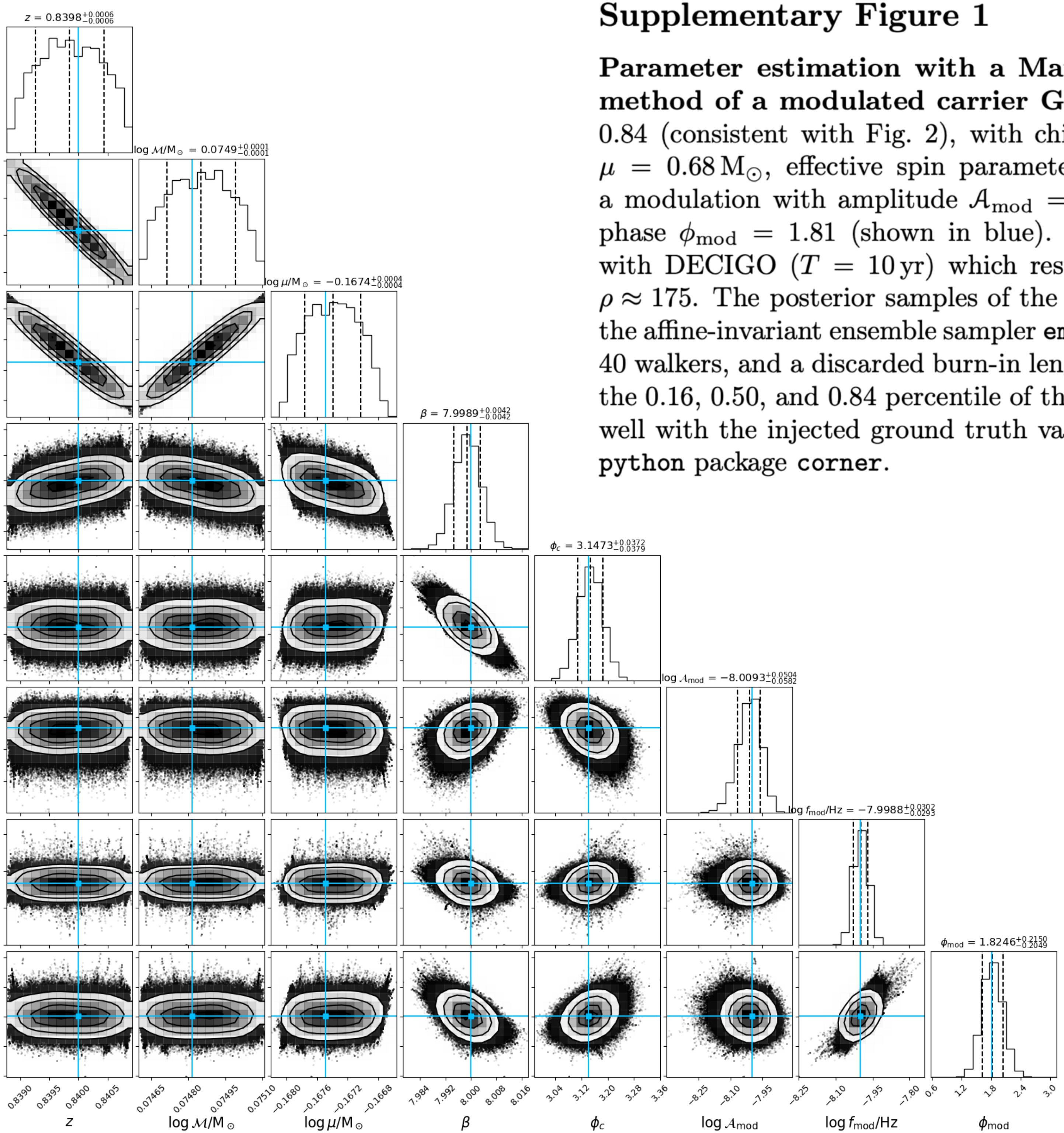

Supplementary Figure 1

Parameter estimation with a Markov chain Monte Carlo (MCMC) method of a modulated carrier GW. We assumed a BNS at redshift $z = 0.84$ (consistent with Fig. 2), with chirp mass $\mathcal{M} = 1.188\,\mathrm{M}_\odot$, reduced mass $\mu = 0.68\,\mathrm{M}_\odot$, effective spin parameter $\beta = 8.0$, and phase $\phi_c = 3.14$ and a modulation with amplitude $\mathcal{A}_\mathrm{mod} = 10^{-8}$, frequency $f_\mathrm{mod} = 10^{-8}\,\mathrm{Hz}$, and phase $\phi_\mathrm{mod} = 1.81$ (shown in blue). We assume that the BNS is observed with DECIGO ($T = 10\,\mathrm{yr}$) which results in an SNR of the carrier signal of $\rho \approx 175$. The posterior samples of the waveform parameters are obtained with the affine-invariant ensemble sampler `emcee` for MCMC for a chain length of 10^6, 40 walkers, and a discarded burn-in length of 5×10^4. The dashed lines indicate the 0.16, 0.50, and 0.84 percentile of the posteriors, which for this system agree well with the injected ground truth values. This plot was generated using the `python` package `corner`.

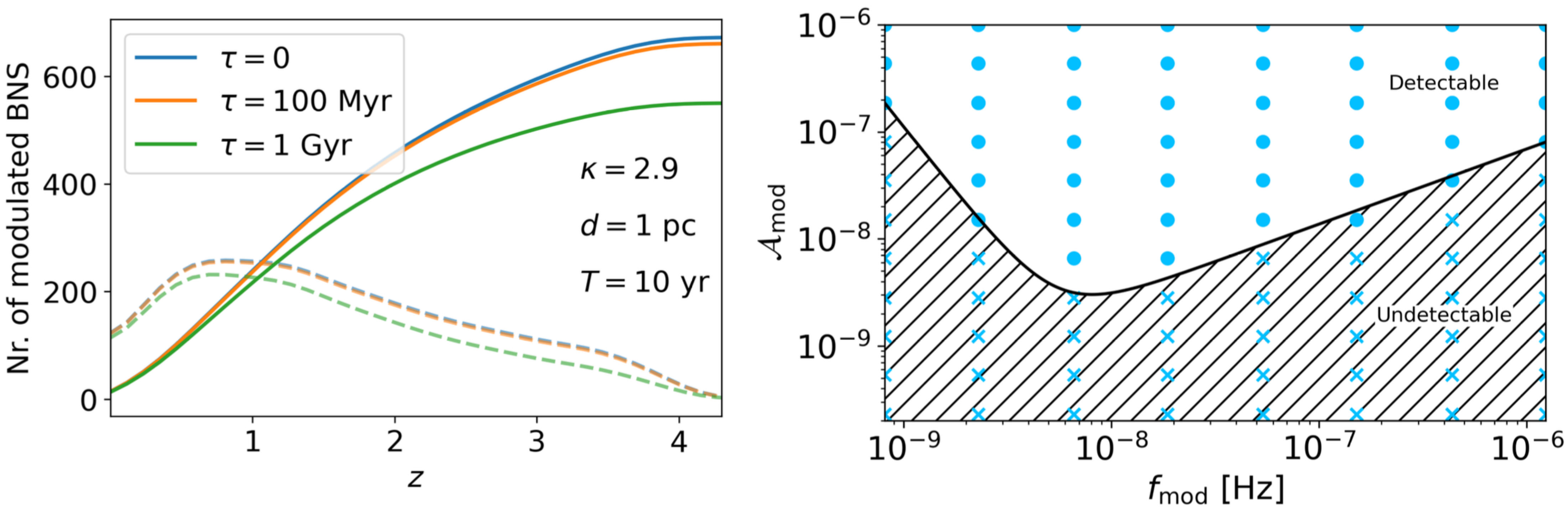

Supplementary Figure 2

Impact of the delay time and results from a grid of MCMC parameter estimations. *Left panel:* We show how the differential (dashed lines) and cumulative (solid lines) distribution of modulated BNSs varies as a function of a constant delay time τ between halo mergers and SMBHB mergers. *Right panel:* Each marker corresponds to a full Bayesian parameter estimation with an MCMC, e.g., as in Supplementary Fig. 1, of a modulated BNS at our exemplary redshift $z = 0.84$. The axes define the amplitude and frequency of the injected modulation. Dots and crosses indicate whether the modulation could be resolved with the MCMC or not, respectively. We define a modulation to be detectable if the one-sigma width of the posterior distribution of $\mathcal{A}_{\rm mod}$ is smaller than the injected value, e.g., as in Supplementary Fig. 1. A sensitivity curve is constructed (solid black line) by fitting a smoothly broken power law of the form $\mathcal{A}_{\rm mod}(f_{\rm mod}) = a[(f_{\rm mod}/f_0)^\alpha + (f_{\rm mod}/f_0)^\beta]$ to the boundary of detectable and undetectable modulations (hatched region) where no modulation could be confidently established. This accommodates different slopes of the sensitivity below and above the frequency at peak sensitivity and is used in Fig. 2.
